# Topological differences of striato‐thalamo‐cortical circuit in functional brain network between premature ejaculation patients with and without depression

**DOI:** 10.1002/brb3.3585

**Published:** 2024-06-07

**Authors:** Xinyue Zhang, Peining Niu, Mengqing Su, Li Zhou, Yunke Huang, Jianhuai Chen, Shaowei Liu

**Affiliations:** ^1^ School of Medicine & Holistic Integrative Medicine Nanjing University of Chinese Medicine Nanjing China; ^2^ Department of Andrology Siyang County Traditional Chinese Medicine Hospital Affiliated to Yangzhou University School of Medicine Suqiang China; ^3^ School of Chinese Medicine, School of Integrated Chinese and Western Medicine Nanjing University of Chinese Medicine Nanjing China; ^4^ Women's Hospital Zhejiang University School of Medicine Zhejiang China; ^5^ Department of Andrology Jiangsu Province Hospital of Chinese Medicine, Affiliated Hospital of Nanjing University of Chinese Medicine Nanjing China; ^6^ Department of Radiology Jiangsu Province Hospital of Chinese Medicine, Affiliated Hospital of Nanjing University of Chinese Medicine Nanjing China

**Keywords:** brain network, depression, graph theoretical analysis, premature ejaculation, resting‐state functional magnetic resonance imaging

## Abstract

**Introduction:**

Premature ejaculation (PE), a common male sexual dysfunction, often accompanies by abnormal psychological factors, such as depression. Recent neuroimaging studies have revealed structural and functional brain abnormalities in PE patients. However, there is limited neurological evidence supporting the comorbidity of PE and depression. This study aimed to explore the topological changes of the functional brain networks of PE patients with depression.

**Methods:**

Resting‐state functional magnetic resonance imaging (rs‐fMRI) data were acquired from 60 PE patients (30 with depression and 30 without depression) and 29 healthy controls (HCs). Functional brain networks were constructed for all participants based on rs‐fMRI data. The nodal parameters including nodal centrality and efficiency were calculated by the method of graph theory analysis and then compared between groups. In addition, the results were corrected for multiple comparisons by family‐wise error (FWE) (*p *< .05).

**Results:**

PE patients with depression had increased degree centrality and global efficiency in the right pallidum, as well as increased degree centrality in the right thalamus when compared with HCs. PE patients without depression showed increased degree centrality in the right pallidum and thalamus, as well as increased global efficiency in the right precuneus, pallidum, and thalamus when compared with HCs. PE patients with depression demonstrated decreased degree centrality in the right pallidum and thalamus, as well as decreased global efficiency in the right precuneus, pallidum, and thalamus when compared to those without depression. All the brain regions above survived the FWE correction.

**Conclusion:**

The results suggested that increased and decreased functional connectivity, as well as the capability of global integration of information in the brain, might be related to the occurrence of PE and the comorbidity depression in PE patients, respectively. These findings provided new insights into the understanding of the pathological mechanisms underlying PE and those with depression.

## Graphical abstract

Premature ejaculation (PE) is often accompanied by abnormal psychological factors, such as depression. We collected resting‐state fMRI data of PE patients with and without depression, as well as healthy controls. Graph theory analysis of functional brain networks underlined the topological changes of pallidum and thalamus as a neuroimaging phenotype for depressive PE.

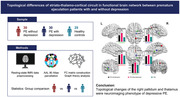

## 1 INTRODUCTION

Premature ejaculation (PE) is the most common sexual dysfunction troubling nearly one‐third of sexually active men globally, which has a substantial negative impact on the quality of life of both the patients and their partners (Raveendran & Agarwal, 2021). Lifelong PE is defined by the International Society of Sexual Medicine (ISSM) as “a male sexual dysfunction characterized by ejaculation that always or nearly always occurs prior to or within about 1 min of vaginal penetration from the first sexual experience” (Althof et al., 2014). Growing studies have provided evidence for multiple explanations of PE etiology. Psychological, endocrine, genetic, neurobiological, and urologic factors are responsible for the occurrence of PE and its development (Coskuner & Ozkan, 2022; Soni et al., 2022). The prevailing perspective has long considered PE as a psychosexual disorder since PE has a complex correlation with psychogenic events like anxiety, depression, guilt, stress, and early sexual experiences (El‐Hamd et al., 2019). Depression is a prevalent and debilitating mood disorder, defined as a persistent feeling of sadness and/or an inability to experience pleasure, with associated deficits in daily function (McCarron et al., 2021). Globally, depression is a threat to 3.8% of the population and is recognized as a primary cause of both mental and physical impairments (Monroe & Harkness, 2022). The existence of a self‐perpetuating downward spiral wherein sexual dysfunction and depression are well acknowledged (Mollaioli et al., 2020), which means the relationship between the above two events is bidirectional (Xia et al., 2016). While depression significantly influences the initiation and persistence of PE, patients with PE tend to have poorer feelings such as self‐estimate, frustration, and anxiety, giving rise to low mood status and depressive disorder (Rajkumar & Kumaran, 2015; Symonds et al., 2003). However, the precise mechanisms underlying the comorbidity between PE and depression remain unclear.

Male sexual behavior is a sequential process of integrated phases, namely, excitement, plateau, orgasm, and ejaculation, as well as resolution. Ejaculation refers to external semen expelling, which contains two independent phases: emission and expulsion. The event of ejaculation requires the cooperative functioning of both autonomic and somatic nervous systems, and cerebral controls (Krassioukov & Elliott, 2017). Neuroimaging research has demonstrated multiple brain regions mediate human sexual function, including the prefrontal cortex, the parietal cortex, and the insula, as well as some limbic structures (e.g., hypothalamus, amygdala, thalamus, cingulate cortex, and septal region) (Calabrò et al., 2019; Cheng et al., 2015). Though not fully elucidated, certain brain regions were found to be intensively active in the mesodiencephalic transition zone, the thalamus, and the parietal cortex during the ejaculation process (Clement & Giuliano, 2016). The brain‐sex bidirectional neuroendocrine axis of sexual and mental interconnected regulation interprets the strong correlation between sexual dysfunction and emotional disorders (Gombert et al., 2021). Depression, as a major mental illness, is identified to have notable variations in brain structure, including the frontal lobe, the parietal lobe, the thalamus, the striatum, and the hippocampus (F.‐F. Zhang et al., 2018). Many of these brain hubs, which form significant emotion regulatory circuits, may also modulate sexual interest, arousal, and behavior (Cheng et al., 2015).

Resting‐state functional magnetic resonance imaging (rs‐fMRI), based on changes in blood oxygen level‐dependent (BOLD) signs, is an effective way to measure spontaneous brain activity. Abnormal functional connectivity was found in the frontal, temporal, and parietal cortex, as well as the limbic system, of PE patients in a resting‐state fMRI study based on machine learning (Z. Xu et al., 2019). Originating from mathematics in the 18th century, graph theory is now an essential tool for systematic and quantitative investigation into complex interconnected networks of nodes and edges including brain network (Sporns, 2011, 2018). Increasing applications in various brain disorders have confirmed that the combination of rs‐fMRI and graph theoretical analysis is beneficial in capturing abnormal topological features of functional brain network (Farahani et al., 2019; Wang et al., 2010). A previous study combining rs‐fMRI and graph theory showed that lifelong PE patients had altered degree centrality in the brain areas involved in the sensory perception, motivational process, and inhibitory control (M. Gao et al., 2021). However, the topological characteristics of the functional brain network of PE patients and those comorbid with depression are limited. In previous studies, we tried to interpret the central pathophysiology of PE and its related emotional disorders (Chen, Yang, et al., 2021; S. Liu et al., 2021; X. Liu et al., 2023; Y. Xu et al., 2021). Decreased functional connectivity was found between the left medial superior frontal gyrus and amygdala of PE patients, which indicated that these brain regions had vital roles in the central control of ejaculation and emotion stability (Y. Xu et al., 2021). In our prior diffusion tensor imaging (DTI) study, the brain networks of PE patients with depression showed abnormal topological organization in the orbitofrontal cortex, which was associated with both sexual and negative emotions (Chen, Yang, et al., 2021).

The objective of this study was to explore changes of topological organization of functional brain networks in PE patients with depression based on rs‐fMRI data using the method of graph theoretical analysis. We hypothesized that PE patients with and without depression might exhibit differences in the topological organization of functional brain networks. Hence, functional brain networks were constructed based on rs‐fMRI data, and then nodal parameters including nodal centrality and efficiency were calculated and compared between groups using the graph theoretical analysis.

## 2 MATERIALS AND METHODS

### 2.1 Participants

The study was approved by the ethical committee of the Jiangsu Province Hospital of Chinese Medicine, Affiliated Hospital of Nanjing University of Chinese Medicine (approval number: 2020NL‐016‐08). The written informed consents were obtained from all participants. The demographic and clinical information of all participants were listed in Table 1.

**TABLE 1:** Demographic and clinical characteristics between groups.

Characteristics	PE with depression (*n* = 30)	PE without depression (*n* = 30)	HC (*n* = 29)	*F*	*p* values
**Age (years)**	34.40 ± 6.28	35.27 ± 7.16	34.66 ± 5.98	0.14	.87
**Educational level (years)**	14.67 ± 2.17	14.43 ± 2.34	15.45 ± 2.50	1.51	.23
**IIEF‐5 (score)**	22.37 ± 0.72	22.47 ± 0.68	22.79 ± 0.86	2.55	.08
**PEDT (score)** ^a^, ^b^	13.70 ± 2.72	14.43 ± 2.74	4.31 ± 2.04	146.69	<.01
**IELT (s)** ^a^, ^b^	87.00 ± 37.25	81.50 ± 40.88	533.79 ± 280.67	74.06	<.01
**HAMD‐17 (score)** ^b^, ^c^	11.67 ± 2.25	3.83 ± 1.68	4.03 ± 1.86	157.10	<.01

*Note*: Multigroup comparisons were carried out by one‐way analysis of variance (ANOVA) test with post hoc contrasts by least‐significant difference (LSD) test.

Abbreviations: HAMD, Hamilton depression scale; HCs, health controls; IELT, intravaginal ejaculation latency time; IIEF, international index of erectile function; PE, premature ejaculation; PEDT, premature ejaculation diagnostic tool.

^a^ Indicated significant differences between PE without depression and HCs.

^b^ Indicated significant differences between PE with depression and HCs.

^c^ Indicated significant differences between two PE groups.

*p *< .05 indicated statistically significant differences among groups.

A total of 60 participants with PE (30 with depression and 30 without depression) were recruited at Jiangsu Province Hospital of Chinese Medicine, Affiliated Hospital of Nanjing University of Chinese Medicine. PE patients were diagnosed by two experienced andrologists following the International Society for Sexual Medicine (ISSM) Guidelines: a short ejaculation latency, a perceived inadequate ability to control the timing of ejaculation, and PE‐related adverse personal consequences (Althof et al., 2010). The inclusion of PE patients: (1) right‐handed and aged between 20 and 45 years old; (2) complained about PE and self‐reported inability to delay ejaculation since the first sexual intercourse on all or nearly all vaginal penetrations; (3) stopwatch measurement of intravaginal ejaculation latency time (IELT) < 1 min; (4) scores of Premature Ejaculation Diagnostic Tool (PEDT) ≥ 11; (5) scores of International Index for Erectile Function (IIEF‐5) > 21; (6) had a stable and heterosexual relationship no less than 6 months with normal sexual desire. The Hamilton Depression Scale‐17 (HAMD‐17) was rated by an experienced psychiatrist on the day of image acquisition to distinguish the presence of depression between the two PE subgroups: patients with a HAMD‐17 score >7 were subdivided into PE patients with depression (*n* = 30), whereas patients with a HAMD‐17 score of ≤7 were subdivided into PE patients without depression (*n* = 30). Additionally, 29 right‐handed healthy controls (HCs) were recruited through advertisement, with matched gender, sexual orientation, age, and educational level with the patients.

All the questionnaires used in our study were validated. PEDT is a commonly used diagnostic tool for PE, consisting of five items. It assigns severity levels as: 8 “no PE,” 9 and 10 “probable PE,” and 11 “PE” (Symonds et al., 2007). The Chinese version demonstrated strong validity and reliability (Huang et al., 2014). The IIEF‐5 scale consists of five items that classified ED into five levels: severe (5–7), moderate (8–11), mild to moderate (12–16), mild (17–21), and no ED (22–25) (Rosen et al., 1997). It has been widely applied in Chinese samples (Niu et al., 2023). HAMD‐17 is a 17‐item scale with scores ranging from 0 to 52 (Hamilton, 1967), where higher numbers indicate more severe depressive symptoms (Zheng et al., 1988).

Exclusion criteria for PE patients and HCs are as follows: (1) any other major psychiatric diseases or major physical diseases; (2) use of psychotropic medicine or medications with potential impact on sexual function; (3) history of head trauma and loss of consciousness; (4) alcohol or substance abuse; and (5) contraindication of MRI scanning.

### 2.2 Imaging acquisition and preprocessing

The MRI data of all participants were acquired on a 3.0T GE MRI scanner. Each participant was required to remain relaxed with their eyes closed and think of nothing in particular. T1‐weighted images were acquired with the following parameters: repetition time (TR) = 7.7 ms; echo time (TE) = 3.1 ms; slice thickness = 1 mm; field of view (FOV) = 256 mm × 256 mm (Althof et al., 2014); matrix = 256 × 256; flip angle (FA) = 12°; number of slices = 160. The rs‐fMRI data were acquired with the following parameters: TR = 2000 ms; TE = 30 ms; slice thickness = 3.5 mm; FOV = 224 mm × 224 mm (Althof et al., 2014); matrix = 80 × 80; FA = 90°; number of slices = 33; number of volumes = 240.

MRI data preprocessing was carried out using Data Processing Assistant for Resting‐State fMRI software (DPARSF) (Version: Advanced; State Key Laboratory of Cognitive Neuroscience and Learning, Beijing Normal University) (Yan et al., 2016). The preprocessing procedure was conducted as follows: (1) Deletion of the first six time points due to the presence of unstable signals and the participants’ adaptation to the scanning conditions; (2) The remaining volumes for slice timing and head‐motion correction (exclusion of participants with more than 2.0 mm head motion or more than 2.0° rotation); (3) Spatial normalization to the Montreal Neurological Institute (MNI, resampled voxel size = 3 mm × 3 mm × 3 mm [Coskuner & Ozkan, 2022]); (4) Smoothing using Gaussian kernel (full‐width at half maximum = 4 mm); (5) Linear detrending and temporal filtering (0.01–0.08 Hz) for minimizing the impacts of high‐frequency noise and low‐frequency drift; (6) Linear regression for nuisance signals (head motion parameters, white matter signal, global signal and cerebral spinal fluid signal).

### 2.3 Network construction and analysis

We constructed the functional brain network as follows: First, we used the automated anatomical labeling (AAL) template (Table S1) to divide the entire brain into 90 regions of interest (ROIs) (Tzourio‐Mazoyer et al., 2002), which were defined as the nodes of the brain network. Second, the time series of all voxels in each ROI were extracted and averaged to obtain the mean time series. Third, Pearson's correlation coefficients between the time series of all ROIs were calculated, representing the functional connectivity strengths between regions, which were defined as the edges of the brain network. A 90 × 90 correlation matrix was then constructed. Fisher's translation was conducted to convert the correlation coefficients to *z* values, after which the functional brain network was formed.

In this study, we applied the GRETNA (http://www.nitrc.org/projects/gretna/) for measuring the topological property of the functional brain network. The threshold values of the correlation matrix were set from 0.05 to 0.4, with the interval of 0.01. In order to identify the differences of the topological organization of functional brain networks between groups, network parameters were collected at the global and local levels. We calculated small‐worldness (σ), which is defined as the ratio of the normalized clustering coefficient (γ) divided by the normalized characteristic path length (λ). Nodal‐level metrics consisted of nodal efficiency (nodal global and nodal local efficiency), degree centrality, and betweenness centrality. Nodal efficiency is regarded as the information transmission ability of a node to the remaining nodes in the network (Ma et al., 2018). Brain regions with higher nodal efficiency are integrated more into the overall network with lower connection cost. Nodal global efficiency is the inverse of the average shortest path length between an individual node and the remaining nodes of the network, while nodal local efficiency describes the global efficiency of the subgraph with the given node's neighbors (H. Chen et al., 2019). Nodal centrality reflects the importance of a node, which is how a node affected or is affected by other parts of the brain network. Degree centrality is equal to nodal degree, which is the most commonly used parameter in graph theory and is calculated as the number of connections of each node, being a sensitive indicator of the central areas in the network. Betweenness centrality is to measure the number or strength of shortest routes that traverse a given node in the network (Rubinov & Sporns, 2010), which helps recognize bridging nodes that carry a significant amount of information flow.

### 2.4 Statistical analysis

The statistical analysis was conducted by the Statistical Package for the Social Sciences (SPSS) (IBM). The normality and homogeneity of variance were tested by the Shapiro–Wilk test and Levene's test, respectively. To compare the group differences in the demographic, clinical characteristics, and nodal metrics, a one‐way analysis of variation (ANOVA) with post hoc contrasts by least‐significant difference (LSD) tests was used. In order to account for the multiple comparisons for nodal parameters involving 90 brain regions, a family‐wise error (FWE) correction was applied to the initial *p*‐value of .05.

## 3 RESULTS

### 3.1 Demographic and clinical characteristics between groups

A total of 89 participants were enrolled in the present study (30 PE with depression, 30 PE without depression, and 29 HCs). The demographic and clinical features of all participants were summarized in Table 1.

### 3.2 Small‐worldness characteristics

**FIGURE 1:**
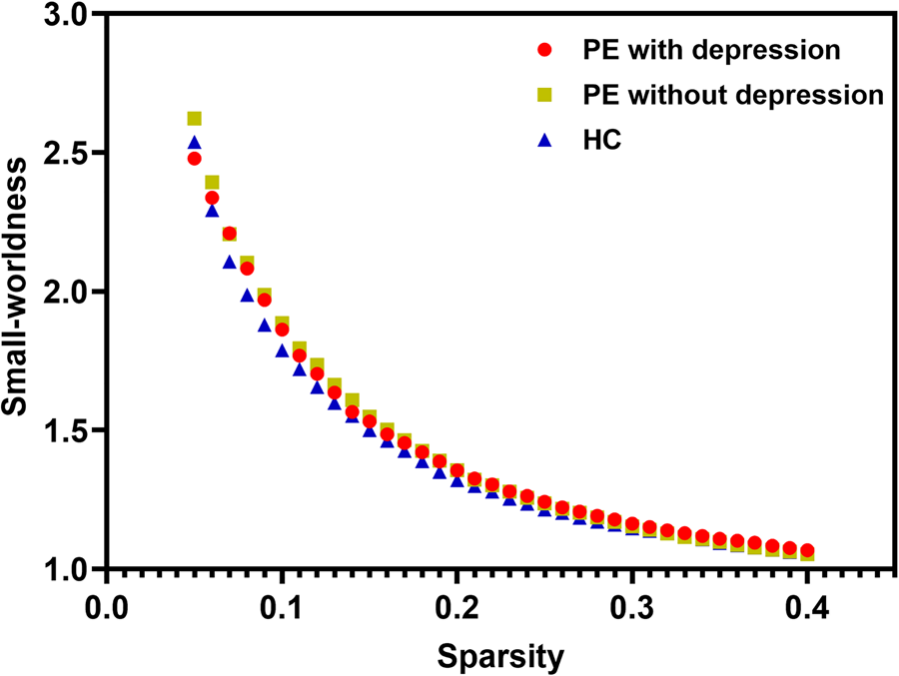
The small‐worldness characteristics of the brain networks. Small‐worldness index σ plotted for network sparsity ranging from 0.05 to 0.4. PE: premature ejaculation; HC: health controls.

Over the sparsity range of 0.05–0.4 (step = 0.01), all groups met the criteria of the small‐world topology (small‐worldness > 1) (Figure 1).

### 3.3 Comparison of nodal degree centrality between groups

**FIGURE 2:**
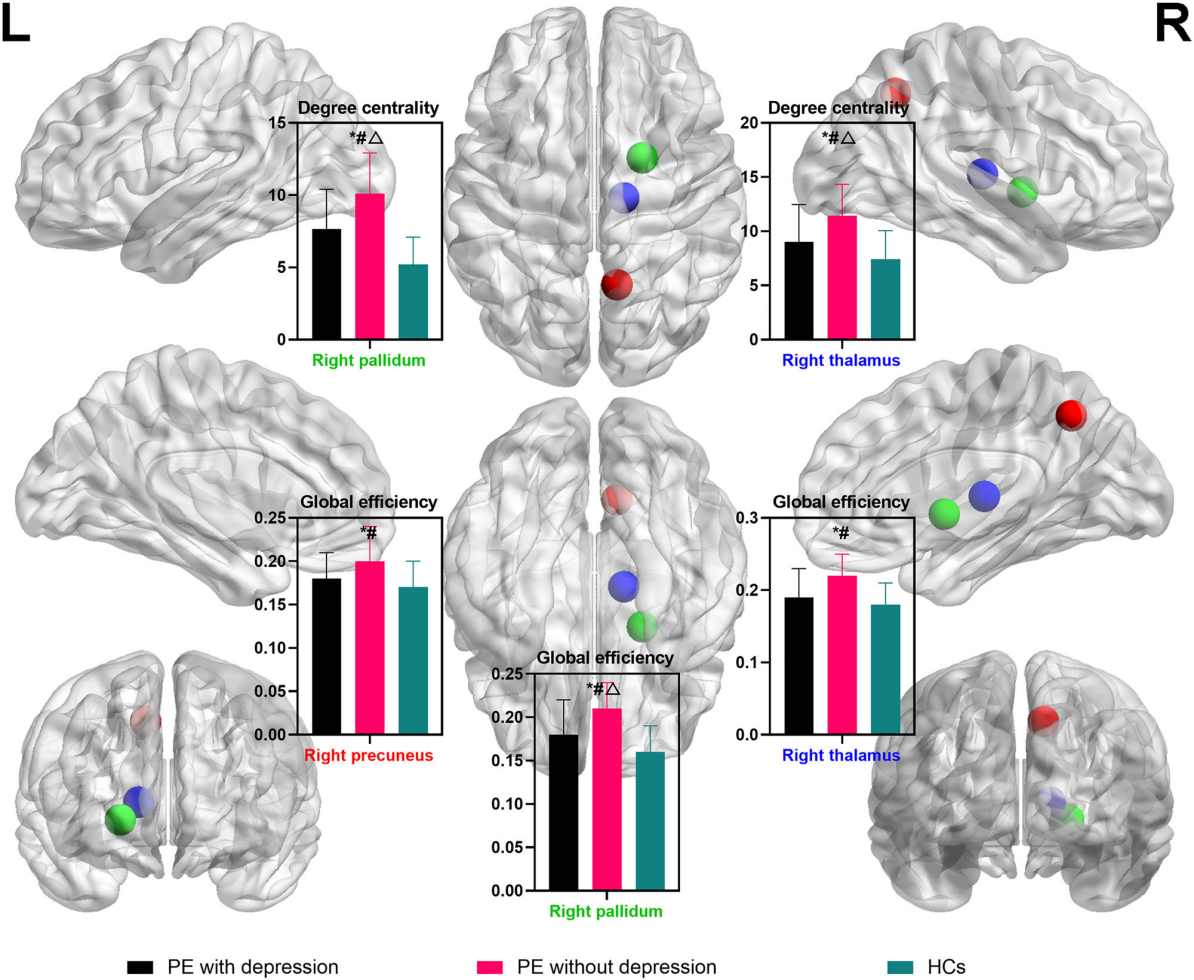
Comparison of nodal parameters between groups. Multigroup comparisons were carried out by one‐way analysis of variance (ANOVA) test with post hoc contrasts by least‐significant difference (LSD) test. ^*^indicated significant differences between two premature ejaculation (PE) groups; ^#^indicated significant differences between PE without depression and health controls (HCs). ^△^indicated significant differences between PE with depression and HCs. In order to account for the multiple comparisons, a family‐wise error (FWE) correction was applied to the initial *p*‐value of .05.

The results of ANOVA analysis indicated that there were significant differences in the nodal degree centrality of the right pallidum and thalamus between groups (survived FWE correction) (Table 2; Figure 2; Table S2).

**TABLE 2:** Comparison of nodal parameters between groups.

Parameters	Brain regions	PE with depression (*n* = 30)	PE without depression (*n* = 30)	HCs (*n* = 29)	*F*	*p* values
**Degree centrality**	Right pallidum^a^, ^b^, ^c^	7.66 ± 2.74	10.09 ± 2.83	5.21 ± 1.89	27.48	<.01
	Right thalamus^a^, ^b^, ^c^	9.01 ± 3.45	11.42 ± 2.91	7.44 ± 2.61	13.05	<.01
**Betweenness centrality**	No significant differences after FEW correction					
**Local efficiency**	No significant differences after FEW correction					
**Global efficiency**	Right precuneus^a^, ^b^	0.18 ± 0.03	0.20 ± 0.04	0.17 ± 0.03	8.59	<.01
	Right pallidum^a^, ^b^, ^c^	0.18 ± 0.04	0.21 ± 0.03	0.16 ± 0.03	17.05	<.01
	Right thalamus^a^, ^b^	0.19 ± 0.04	0.22 ± 0.03	0.18 ± 0.03	10.66	<.01

*Note*: Multigroup comparisons were carried out by one‐way analysis of variance (ANOVA) test with post hoc contrasts by least‐significant difference (LSD) test. In order to account for the multiple comparisons, a family‐wise error (FWE) correction was applied to the initial *p*‐value of .05.

Abbreviations: PE, premature ejaculation; HCs, health controls.

^a^ Indicated significant differences between two PE groups.

^b^ Indicated significant differences between PE without depression and HCs.

^c^ Indicated significant differences between PE with depression and HCs.

The post hoc contrasts showed that both PE patients with and without depression had an increased nodal degree in the right pallidum and thalamus when compared with HCs. Moreover, PE patients with depression showed decreased nodal degree in the right pallidum and thalamus when compared to those without depression (Table 2; Figure 2; Table S2).

### 3.4 Comparison of nodal betweenness centrality between groups

The results of the ANOVA analysis indicated that there were no significant differences in the nodal betweenness centrality between groups after FWE correction (Table S2).

### 3.5 Comparison of nodal local efficiency between groups

The results of the ANOVA analysis indicated that there were no significant differences in the nodal local efficiency between groups after FWE correction (Table S2).

### 3.6 Comparison of nodal global efficiency between groups

The results of ANOVA analysis indicated that there were significant differences in the global efficiency of the right precuneus, pallidum, and thalamus in the nodal global efficiency between groups (survived FWE correction) (Table 2; Figure 2; Table S2).

The post hoc contrasts showed that PE patients with depression had increased global efficiency in the right pallidum when compared with HCs. PE patients without depression showed increased global efficiency in the right precuneus, pallidum, and thalamus when compared with HCs. PE patients with depression demonstrated decreased global efficiency in the right precuneus, pallidum, and thalamus when compared to those without depression (Table 2; Figure 2; Table S2).

## 4 DISCUSSION

In this study, we explored the topological organization of the functional brain networks between PE patients with or without depression and HCs based on rs‐fMRI using the method of graph theoretical analysis. The main findings were as follows: (1) PE patients with depression showed increased degree centrality in the right pallidum and thalamus, as well as increased global efficiency in the right pallidum when compared with HCs; (2) PE patients without depression exhibited increased degree centrality in the right pallidum and thalamus, as well as increased global efficiency in the right precuneus, pallidum, and thalamus when compared with HCs; and (3) PE patients with depression demonstrated decreased degree centrality in the right pallidum and thalamus, as well as decreased global efficiency in the right precuneus, pallidum, and thalamus when compared to those without depression. These findings further interpreted the topological alterations of brain networks in PE patients and those with depression.

Sexual behavior is a combined physical and psychological action that is mediated by the central neural system. Both cortical and subcortical regions, which form interconnected neural networks, are responsible for the sexual response cycle, including sexual desire, excitement, and orgasm (Baird et al., 2007; Poeppl et al., 2016). Ejaculation, a vital part of male sexual behavior, is controlled by brain areas involved in the sensory and motor processing (Alwaal et al., 2015). PE patients without depression had increased global efficiency in the right precuneus compared with HCs. The precuneus is located in the medial parietal cortex and is one of the most connected hub regions in the brain (Gusnard et al., 2001), which has wide‐ranging connections with cortical and subcortical regions (Cavanna & Trimble, 2006). Precuneus is the core component of three distinct brain networks including the default mode network (DMN) (Dadario & Sughrue, 2023; Margulies et al., 2009), and has been identified to play a central role in higher‐order behavioral functions, combining both external and internal information (Smitha et al., 2017; Utevsky et al., 2014). Many studies have demonstrated its involvement in self‐referential processes, episodic memory, and visuo‐spatial imagery (Jitsuishi & Yamaguchi, 2021; Sajonz et al., 2010). In a positron emission tomography study, healthy participants under manual penile stimulation without visual input showed activation in the precuneus, which might be attributed to the visual imagery in the physiological process of ejaculation (Holstege et al., 2003). A similar neuroimaging study demonstrated that lifelong PE patients showed increased degree centrality in the bilateral precuneus (M. Gao et al., 2021). PE patients also had increased long‐range functional connectivity density in the bilateral precuneus (Lu et al., 2018). Meanwhile, lifelong PE patients exhibited higher voxel‐mirrored homotopic connectivity values in the precuneus (Feng et al., 2021). Our findings aligned with the previously reported altered functional connectivity in the precuneus of PE patients, which deepened the current understanding of its role in the sexual‐related self‐referential processing. The observed overactivation of the precuneus located in DMN might indicate that individuals with PE exhibit heightened sensitivity to erotic stimuli (Chen et al., 2021). The increased global efficiency in the precuneus of PE patients reflected the hyperactive functional integration, which decreased the threshold and contributed to rapid ejaculation.

Many neuroimaging studies have reported the thalamus's role in mediating sexual control as an integration hub for desire, arousal, and orgasm processes, as well as the major component in the reward system (Calabrò et al., 2019; M. Gao et al., 2022; Holstege et al., 2003; Lu et al., 2020; Mukku et al., 2022). As a crucial mediator in relaying sensory signals, the thalamus receives output from lumbar spinothalamic cells (LSt cells) from spinal cord, giving supraspinal‐level control of ejaculation (McKenna, 2022). In neuroimaging research, somatosensory brain areas, including the thalamus, were reported to be activated physiologically during visual erotic stimulation (Park et al., 2001) and the ejaculation process (Holstege et al., 2003). The right thalamus showed a positive correlation with the degree of penile tumescence (Moulier et al., 2006). Higher inter‐hemisphere thalamic interaction represented the enhanced sensorimotor processing to compensate for other defective brain regions (Feng et al., 2021). Likewise, the increased axial diffusivity of the right posterior thalamic radiation supported the progression of accelerated sensory processing in PE (M. Gao et al., 2018), which might explain the higher thalamic global efficiency in our present study. A dual control model of sexual response states that sexual behavior is mediated by the balance of excitation and inhibition (Janssen & Bancroft, 2023). Both structural and functional damage to thalamus highlighted a deficiency in its ejaculatory inhibition control (M. Gao et al., 2022; S. Gao et al., 2023). The higher propensity for sexual excitation and stronger reaction to sexual stimuli were found in PE patients (Ventus & Jern, 2021). Therefore, we also speculated subsequently elevated sexual inhibitory demand in the mechanism of rapid ejaculation, which is presented by enhanced nodal topological properties. Thalamus also has complex connectivity with the pallidus, which is one of the major components of striatum in the basal ganglia, through dopaminergic pathways (Mendez & Shapira, 2013). As a major relay station for cortical input, the thalamus plays a crucial role in the cortico‐striato‐thalamo‐cortical circuit (CSTC) (Metzger et al., 2013), which is implicated in movement reward processing and emotion control (Heatherton & Wagner, 2011; Rădulescu et al., 2017). From the cortex, the CSTC circuit extends to the striatum, then to the thalamus via the globus pallidus, and finally back to the cortex. The fibers projecting from the pallidus to the thalamus were found to affect sexual drive (Baird et al., 2007; Mendez et al., 2004). Our previous DTI study identified abnormal nodal efficiency in the right pallidum and thalamus, representing that decreased segregation of these regions might facilitate ejaculation (J. Chen et al., 2020). Likewise, a structural MRI study demonstrated increased structural covariance between the striatum and thalamus, which showed potential higher sensitivity to sexual stimulation in PE patients (Wu et al., 2022). A recent study observing the response of PE patients to both sexual and nonsexual rewards suggested enhanced incentive motivation and hedonic impact of sexual stimuli, which highlighted an allostatic shift in the reward processing pathways of PE patients (Li, Wang, et al., 2023). Based on our findings, the higher functional connectivity and increased global integration of information might again reinforce the abnormalities of PE patients in sensory and emotional processing, controlling sexual inhibition, and reacting to reward‐related information (Wu et al., 2022).

In addition, topological alterations were also found in the right precuneus, pallidum, and thalamus in PE patients with depression compared with others without depression. Patients with PE often exhibit disrupted psychological states in clinical settings. The presence of impaired self‐esteem and loss of interest in individuals with depression can lead to negative feelings such as frustration or fear, which may be detrimental to male sexual behaviors and result in a vicious cycle. The correlation between psychiatric diseases, including depression and PE, has been well discussed etiologically and theoretically (J. Gao et al., 2013; T. A. O. Liu et al., 2019). Given that serotonin (5‐hydroxytrypatamine [5‐HT]) mediated inhibitory control of the ejaculatory process, serotonergic pathway impairment was implicated in the central mechanism of PE development (Giuliano & Clement, 2006). Decreased serotonin at the synaptic level was associated with the hyperactivity of sympathetic nerve system and ejaculatory reflex, serving as another neurological factor (Li et al., 2023). Thus, selective serotonin reuptake inhibitors (SSRIs) were effective by blocking serotonin transports and elevating serotonin at synaptic gap (Sathianathen et al., 2021). Central serotonin transmission also innervated various cognitive emotion brain regions (Wolf et al., 2018), suggesting the potential interplay between PE and emotions. It was reported that anxiety or other negative states were accompanied by premature sympathetic trigger and monitoring distraction, which led to PE (Buvat, 2011). PE patients with anxiety demonstrated abnormal prefrontal cortex activation, with abnormal inhibitory control and increased attention to external stimuli, facilitating ejaculation (Liu et al., 2021). As the central component of emotion‐related limbic system (Šimić et al., 2021), the amygdala was involved in regulating PE (Geng et al., 2021) and modifying subsequent negative emotions (Y. Xu et al., 2021). Other studies suggested that the association between cognitive, emotional disorders and PE could potentially be attributed to the regulation of the reward system (Barata, 2017; Ha et al., 2019). However, the neurological evidence for depressive PE remained inadequate. Our previous DTI study based on graph analysis first reflected that the comorbidity of PE and depression had a structural basis, and the orbitofrontal cortex was a crucial region (Chen et al., 2021).

It was noteworthy in our current study that PE patients with or without depression both presented higher degree centrality and global efficiency in the right pallidum, as well as increased degree centrality in the right thalamus, when compared with HCs. However, the global efficiency of right pallidum and thalamus was found to decrease in depressive PE patients in comparison with non‐depressive patients, which implied reduced local capacity of information access. The compromised DMN and frontal‐subcortical circuits in depressive individuals were reported in numerous neuroimaging studies, which agreed with our findings (Korgaonkar et al., 2014; Yun & Kim, 2021). Giving the top‐down control over the CSTC circuit (Kim et al., 2019), the pallidus is associated with the motor control, reward, emotional processing, and motivation (Ceravolo et al., 2021; Howell et al., 2016; Ricciardi et al., 2023). A voxel‐based morphometry study found subthreshold depressive patients had significantly decreased gray matter volume in the bilateral pallidus, which correlated with the severity of depression (J. Li et al., 2017). Likewise, a recent rs‐fMRI study demonstrated that disrupted functional connectivity between the bilateral pallidum was the most important biomarker for subthreshold depression, and it had the strongest relevance to rewards and anhedonia (Sato et al., 2023). Moreover, the estimated increased brain iron deposits in the right pallidus indicated potential cellular damage and death, which were notably linked with attention deficiency (Jakary et al., 2023). A structural MRI study reported that structural and functional abnormalities of the CSTC circuit contribute to the onset of somatic symptoms of depression patients (Sun et al., 2023). The CSTC circuit was at the core of cognitive processes and goal‐directed behavior, triggering various psychiatric disorders (Peters et al., 2016). It was identified that obsessive‐compulsive disorder patients exhibited atypical CSTC function, with the thalamus and pallidum recognized as pivotal brain regions (Kong et al., 2020). Within the limbic system, the thalamus was engaged in modulating anxiety and stress as a coordination center, alongside the prefrontal cortex and the amygdala (Kenwood et al., 2022; X. Zhang et al., 2019). Besides the classical nodes, the striatum was also considered a crucial hub in the anxiety circuit (Lago et al., 2017). The relationship between PE and emotion is intricate, necessitating further exploration beyond depression (Barone et al., 2022). Of note, the topologically abnormal brain regions in our findings all concentrated on the right hemisphere, which highlighted the hemispheric specialization in sexual behavior and mental processes. The right‐dominant hemispheric asymmetry was associated with orgasm (Suffren et al., 2011) and cognitive phenomenology processing of depression (Hecht, 2010).

The integrative findings implied that the hyperfunction of the pallidum and thalamus in PE might also play a compensatory role in regulating emotion positively, yet could not improve PE symptoms. The comorbid depression altered the brain function of PE patients, accompanied by the deficiency in emotional control. Compared to HCs, PE patients with depression exhibited higher brain parameters, which might be attributed to increased comorbid neuropathological burden and widespread intrinsic brain activity, which could not be solely explained by a positive position (Beason‐Held et al., 2022). The neurofunctional changes in the three brain regions might further elucidate the pathophysiological basis of depression and PE, and the pallidum and thalamus, notably, were likely to play central roles in the context of depression and PE. Our findings reinforced the neurobiological basis underlying PE and emotions, transcending mere behavioristic views. The alterations in the pallidum and thalamus might serve as a specific neuroimaging marker to distinguish depressive PE, which hold promise for novel explorations in therapeutic strategies targeting PE with comorbid depression.

There were several limitations to our study. Firstly, the sample scale of our cross‐sectional study is relatively small. The causal relationship between PE and depression remained unclear. More longitudinal studies with larger cohorts should be carried on for more robust and mechanistic conclusions. Secondly, our present study focused on the interplay between depression and PE. Other psychological factors (such as anxiety and stress) can also influence male sexual behavior and should be included to provide a more comprehensive understanding. Thirdly, we used a scale instead of a psychiatry assessment for depression evaluation, and we set relatively lenient inclusion criteria. Factor or subgroup analysis based on depressive severity was expected to be done in the future. Lastly, more methodological optimization, such as eliminating the impact of physiological noise on MRI data and applying different preprocessing tools (Esteban et al., 2019; Waller et al., 2022), was considered for further investigation.

## 5 CONCLUSION

In summary, based on graph theory, this rs‐fMRI study provided support for functional impairments within the components of DMN and cortical‐subcortical circuits of PE. Abnormal topological organization was observed in the precuneus, thalamus, and pallidus of patients with PE. The between‐group differences underlined the roles of the right pallidus and thalamus as pivotal neuroimaging markers for depressive PE. The increased and decreased functional connectivity, as well as the capability of global integration of information in the brain, might be related to the occurrence of PE and the comorbidity depression in PE patients, respectively.

## AUTHOR CONTRIBUTIONS

**Xinyue Zhang**: Writing—original draft; writing—review and editing; methodology; formal analysis. **Peining Niu**: Writing—review and editing; methodology; formal analysis. **Mengqing Su**: Data curation; formal analysis. **Li Zhou**: Data curation; formal analysis. **Yunke Huang**: Data curation; formal analysis. **Jianhuai Chen**: Conceptualization; investigation; funding acquisition; methodology; formal analysis; data curation; supervision; validation; visualization; project administration; writing—original draft; writing—review and editing. **Shaowei Liu**: Conceptualization; investigation; funding acquisition; writing—original draft; methodology; validation; visualization; writing—review and editing; formal analysis; project administration; data curation; supervision.

## CONFLICT OF INTEREST STATEMENT

The authors declare no conflicts of interest.

### PEER REVIEW

The peer review history for this article is available at https://publons.com/publon/10.1002/brb3.3585.

## Supporting information

Supplement 1. Cortical and sub‐cortical regions as anatomically defined in the AAL template and their corresponding abbreviations
Supplement 2. Comparison of nodal parameters between groups

## Data Availability

The data that support the findings of this study are available from the corresponding author upon reasonable request.
