# Supplementary material for: Topological differences of striato‐thalamo‐cortical circuit in functional brain network between premature ejaculation patients with and without depression
Source: Brain Behav. 2024 Jun 7;14(6):e3585. doi: 10.1002/brb3.3585 (PMC11161395; doi:10.1002/brb3.3585)
Supplement: Supplementary file 1 — Supplement 1. Cortical and sub‐cortical regions as anatomically defined in the AAL template and their corresponding abbreviations Supplement 2. Comparison of nodal parameters between groups [file BRB3-14-e3585-s001.docx]

**Supplement 1. Cortical and sub-cortical regions as anatomically defined in the AAL template and their corresponding abbreviations**

| **Region name** | **Abbreviation** |
| --- | --- |
| Precentral gyrus | PreCG |
| Postcentral gyrus | PosCG |
| Rolandic operculum | ROL |
| Superior frontal gyrus, dorsolateral | SFGdor |
| Superior frontal gyrus, orbital | SFGorb |
| Superior frontal gyrus, medial | SFGmed |
| Superior frontal gyrus, medial orbital | SFGmedorb |
| Middle frontal gyrus | MFG |
| Middle frontal gyrus, orbital | MFGorb |
| Inferior frontal gyrus, opercular | IFGoper |
| Inferior frontal gyrus, triangular | IFGtri |
| Inferior frontal gyrus, orbital | IFGorb |
| Supplementary motor area | SMA |
| Olfactory cortex | OLF |
| Gyrus rectus | GRE |
| Paracentral lobule | PCL |
| Heschl gyrus | HES |
| Superior temporal gyrus | STG |
| Middle temporal gyrus | MTG |
| Inferior temporal gyrus | ITG |
| Superior parietal gyrus | SPG |
| Inferior parietal, but supramarginal and angular gyri | IPL |
| Supramarginal gyrus | SMG |
| Angular gyrus | ANG |
| Precuneus | PCUN |
| Calcarine fissure and surrounding cortex | CAL |
| Cuneus | CUN |
| Lingual gyrus | LING |
| Superior occipital gyrus | SOG |
| Middle occipital gyrus | MOG |
| Inferior occipital gyrus | IOG |
| Fusiform gyrus | FFG |
| Anterior cingulate and paracingulate gyri | ACG |
| Median cingulate and paracingulate gyri | DCG |
| Posterior cingulate gyrus | PCG |
| Hippocampus | HIP |
| Parahippocampal gyrus | PHG |
| Temporal pole: superior temporal gyrus | TPOstg |
| Temporal pole: middle temporal gyrus | TPO |
| Amygdala | AMY |
| Caudate nucleus | CAU |
| Lenticular nucleus, putamen | PUT |
| Lenticular nucleus, pallidum | PAL |
| Thalamus | THA |
| Insula | INS |

AAL: Automated anatomical labeling

**Supplement 2. Comparison of nodal parameters between groups**

| **Parameters** | **Brain regions** | **PE with depression**  **(n=30)** | **PE without depression**  **(n=30)** | **HC**  **(n=29)** | ***F*** | ***P* values** |
| --- | --- | --- | --- | --- | --- | --- |
| **Degree** | SFGmed.L^△^ | 7.66±4.02 | 9.43±3.62 | 9.96±3.46 | 3.13 | 0.049 |
|  | IOG.R^#△^ | 6.57±3.20 | 6.69±3.71 | 9.37±4.08 | 5.42 | 0.0061 |
|  | SMG.L^*#^ | 11.76±3.77 | 14.54±4.11 | 12.21±4.65 | 3.80 | 0.026 |
|  | ANG.L^*#^ | 12.11±3.69 | 14.48±4.97 | 11.77±3.56 | 3.80 | 0.026 |
|  | PCUN.L^#^ | 8.72±3.50 | 10.47±3.85 | 7.37±2.86 | 6.04 | 0.0035 |
|  | PCUN.R^*#^ | 6.88±2.80 | 8.81±3.41 | 6.06±2.05 | 7.45 | 0.0010 |
|  | PAL.L^#^ | 7.98±3.26 | 9.68±3.95 | 6.80±2.98 | 5.29 | 0.0068 |
|  | **PAL.R**^*#△^ | **7.66±2.74** | **10.09±2.83** | **5.21±1.89** | **27.48** | **<0.01** |
|  | THA.L^#^ | 9.65±3.49 | 10.82±3.71 | 8.58±2.89 | 3.24 | 0.044 |
|  | **THA.R**^*#△^ | **9.01±3.45** | **11.42±2.91** | **7.44±2.61** | **13.05** | **<0.01** |
|  | STG.R^*#^ | 2.13±1.91 | 3.82±2.81 | 1.94±2.10 | 5.99 | 0.0037 |
| **Betweenness** | ORBsup.R^*#^ | 12.30±15.54 | 4.83±6.77 | 12.29±12.71 | 3.71 | 0.028 |
|  | MOG.L^*△^ | 26.75±23.38 | 17.35±18.14 | 14.78±10.17 | 3.59 | 0.032 |
|  | ANG.R^#^ | 30.88±24.50 | 26.45±27.53 | 45.24±31.94 | 3.59 | 0.032 |
|  | PAL.R^#△^ | 18.67±26.95 | 18.63±17.11 | 6.94±7.91 | 3.69 | 0.029 |
| **Local efficiency** | SFGdor.R^*#^ | 0.26±0.05 | 0.30±0.08 | 0.25±0.06 | 5.12 | 0.0080 |
|  | ROL.R^*#^ | 0.19±0.08 | 0.24±0.08 | 0.19±0.11 | 3.24 | 0.044 |
|  | INS.R^*^ | 0.28±0.05 | 0.32±0.05 | 0.30±0.05 | 3.89 | 0.024 |
|  | PHG.L^*#^ | 0.26±0.07 | 0.31±0.09 | 0.25±0.08 | 5.43 | 0.0060 |
|  | PHG.R^*#^ | 0.26±0.06 | 0.30±0.08 | 0.25±0.07 | 3.93 | 0.023 |
|  | PCUN.R^#^ | 0.28±0.07 | 0.31±0.07 | 0.26±0.07 | 3.57 | 0.032 |
|  | CAU.R^*^ | 0.27±0.06 | 0.31±0.06 | 0.28±0.06 | 3.77 | 0.027 |
|  | STG.R^*#^ | 0.16±0.10 | 0.22±0.11 | 0.13±0.10 | 5.97 | 0.0037 |
|  | TPOmid.R^#^ | 0.26±0.08 | 0.29±0.09 | 0.23±0.09 | 3.71 | 0.028 |
| **Global efficiency** | ROL.R^*#^ | 0.11±0.05 | 0.14±0.04 | 0.11±0.05 | 3.80 | 0.026 |
|  | REC.L^#^ | 0.20±0.04 | 0.22±0.05 | 0.19±0.04 | 3.20 | 0.046 |
|  | PHG.L^#^ | 0.16±0.04 | 0.17±0.04 | 0.15±0.04 | 3.85 | 0.025 |
|  | PHG.R^#^ | 0.17±0.03 | 0.18±0.04 | 0.16±0.04 | 3.76 | 0.027 |
|  | SMG.L^*#^ | 0.21±0.04 | 0.25±0.05 | 0.22±0.05 | 3.98 | 0.022 |
|  | ANG.L^*#^ | 0.22±0.04 | 0.25±0.05 | 0.22±0.04 | 4.12 | 0.020 |
|  | PCUN.L^*#^ | 0.19±0.04 | 0.21±0.04 | 0.18±0.03 | 7.40 | 0.0011 |
|  | **PCUN.R**^*#^ | **0.18±0.03** | **0.20±0.04** | **0.17±0.03** | **8.59** | **<0.01** |
|  | CAU.L^*^ | 0.19±0.04 | 0.22±0.04 | 0.20±0.04 | 3.80 | 0.026 |
|  | CAU.R^*^ | 0.18±0.04 | 0.21±0.04 | 0.20±0.04 | 4.29 | 0.017 |
|  | PAL.L^*#^ | 0.18±0.04 | 0.21±0.04 | 0.17±0.04 | 5.16 | 0.0076 |
|  | **PAL.R**^*#△^ | **0.18±0.04** | **0.21±0.03** | **0.16±0.03** | **17.05** | **<0.01** |
|  | THA.L^#^ | 0.20±0.04 | 0.21±0.04 | 0.19±0.04 | 3.65 | 0.030 |
|  | **THA.R**^*#^ | **0.19±0.04** | **0.22±0.03** | **0.18±0.03** | **10.66** | **<0.01** |
|  | STG.R^*#^ | 0.09±0.04 | 0.12±0.05 | 0.08±0.05 | 6.32 | 0.0027 |

PE: premature ejaculation; HCs: health controls. Multigroup comparisons were carried out by one-way analysis of variance (ANOVA) test with post hoc contrasts by least-significant difference (LSD) test. *indicated significant differences between two PE groups; #indicated significant differences between PE without depression and HCs. △indicated significant differences between PE with depression and HCs. In order to account for the multiple comparisons, a family-wise error (FWE) correction was applied to the initial P-value of 0.05. Bold font represented brain regions survived FWE correction while non-bold font represented brain regions did not survive FWE correction.
